# Evaluation of microaneurysms as predictors of therapeutic response to anti-VEGF therapy in patients with DME

**DOI:** 10.1371/journal.pone.0277920

**Published:** 2022-11-28

**Authors:** Makoto Hatano, Fumiaki Higashijima, Takuya Yoshimoto, Tadahiko Ogata, Manami Ohta, Yuka Kobayashi, Makiko Wakuta, Ryoji Yanai, Kazuhiro Kimura

**Affiliations:** Department of Ophthalmology, Yamaguchi University Graduate School of Medicine, Ube City, Yamaguchi, Japan; Tsukazaki Hospital, JAPAN

## Abstract

Administration of intravitreal anti-vascular endothelial growth factor (anti-VEGF) therapy is the first-line therapy for diabetic macular oedema (DME). However, some patients show no or insufficient response to repeated anti-VEGF injections. Therefore, it is necessary to identify factors that can predict this resistance against anti-VEGF treatment. Presence of microaneurysms (MAs) is a predictor of the development and progression of DME, but its relationship with the treatment response to the anti-VEGF agents is not well known. Therefore, we aimed to elucidate the relationship between the distribution of MAs and the response to anti-VEGF therapy in patients with DME. The number of MAs was measured before anti-VEGF therapy in each region using fluorescein angiography, indocyanine green angiography (IA), and optical coherence tomography angiography. Patients with DME were divided into the responder and non-responder groups after three loading phases. Differences in the distribution of MAs between the groups were investigated. Pre-treatment IA revealed more MAs in the nasal area in the non-responder group than in the responder group (10.7 ± 10.7 and 5.7 ± 5.7, respectively, in the nasal macula) (1.4 ± 2.1 and 0.4 ± 0.7, respectively, in the nasal fovea). Whereas, pre-treatment FA and OCTA could not reveal significantly difference between the groups. Detection of MAs in the nasal macula using pre-treatment IA may indicate resistance to anti-VEGF therapy. We recommend the clinicians confirm the presence of MAs in the nasal macula, as shown by IA, as a predictor of therapeutic response to anti-VEGF therapy in patients with treatment naive DME.

## Introduction

Diabetic macular oedema (DME) is a disease that causes vision loss in patients with diabetic retinopathy. DME is a common complication of diabetic retinopathy caused by intraretinal fluid accumulation in the macular area [1]. Current treatments for DME include focal laser photocoagulation, steroid injection, vitrectomy, and intravitreal injection of anti-vascular endothelial growth factor (anti-VEGF). Anti-VEGF therapy is usually the first-line treatment for DME, and it greatly improves visual acuity [2]. A randomised clinical trial showed that best-corrected visual acuity (BCVA) improved by approximately 8–12 letters from baseline after 24 weeks of anti-VEGF therapy and that BCVA and central subfield thickness improved by 10.0–12.8 letters and 30.0–41.6%, respectively, after treatment with anti-VEGF and focal/grid laser at the 2-year visit [3]. Anti-VEGF drugs have an intensive effect on patients with DME; however, some patients show poor or no response to anti-VEGF drugs despite repeated injections. Specifically, previous studies reported that 18–30% of DME eyes show resistance to anti-VEGF therapy [4, 5]. Therefore, it is necessary to identify predictive factors of resistance to anti-VEGF agents before treatment.

A microaneurysm (MA) is a histopathological dilation of capillaries associated with the loss of pericytes and endothelial cells or thickening of the basement membrane [6, 7]. The relationship between MA and DME has been reported as follows: MA turnover is a predictor of DME progression [8]; the presence of an MA within one optic disc diameter of the fovea is a predictor of DME progression [9]; and analysis of MA formation rate using a software could help identify patients with a higher risk of developing DME [10]. In other words, these studies indicate that MA is a predictor of DME. MAs are localised mainly in the deeper part of the inner retinal capillary plexus and cause retinal thickening and focal or diffuse macular oedema [11]. Leakage from MAs and the number of MAs can be risk factors of visual loss [12, 13].

MAs can be detected using fundus photography, fluorescein angiography (FA), indocyanine green angiography (IA), and optical coherence tomography angiography (OCTA). Because the light emitted by the fluorescein dye excitation is of a short wavelength, it does not penetrate the retinal pigment epithelium (RPE) and shows retinal blood vessels with good contrast [14]. However, the light emitted by the excitation of indocyanine green dye is infrared, penetrates the RPE, and can show choroidal vasculature [15]. In addition, IA stays in blood vessels by binding more strongly to proteins in the blood than does FA [16]. OCTA is an angiographic method that uses the Doppler effect to capture vascular images with OCT and does not require dye injection [17]. There are various reports on the characteristics of MAs in patients with DME as detected by angiography. FA is more sensitive at detecting MAs than is IA or OCTA, and the former can detect leakage from MAs, the cause of retinal oedema [11, 18]. IA can easily detect large MAs with high clinical significance [19]. MA-detection sensitivity of OCTA is same as that of IA and FA [18, 20], and the former method can detect MAs in the retinal capillary plexus by stratified analysis [21]. However, OCTA has some limitations associated with artefacts and segmentation errors when evaluating an oedematous retinal layer in patients with DME [22].

A few studies have indicated the relationship between MA and the therapeutic response to anti-VEGF drugs. The number of MAs in the deep capillary plexus on OCTA was reported to be high in treatment-resistant eyes [23]. The number of MAs detected using late-phase IA was high in patients experiencing DME recurrence after anti-VEGF therapy [24]. In this study, we examined the number and distribution of MAs using each angiography technique before anti-VEGF therapy in patients with DME. We then investigated the correlation between the characteristics of MA and resistance to anti-VEGF therapy in the context of DME.

## Materials and methods

This report was derived from a prospective multicentre clinical study [Ranibizumab exploratory study on the evaluation of the local laser combination therapy to the non-reactive group to the diabetic macular oedema patients (RELAND study)] [UMIN000024208] [jRCTs061180035]. The inclusion criteria of the study were as follows: eyes must show definite retinal thickening due to DME as revealed by clinical examination using techniques such as slit-lamp examination and OCT, and at least one eye of each patient must meet all of the inclusion criteria and none of the exclusion criteria (Table 1).

**Table 1:** Inclusion and exclusion criteria.

Inclusion criteria	Exclusion criteria
Willingness and the ability to provide signed informed consent	History of any anti-VEGF treatment for DME
Age ≥ 20 years	Persistent macula edema for ≥12 months
Type 1 or type 2 diabetes	Macular edema considered to be due to a cause other than diabetic macular edema
HbA1C of < 10% within two months prior to the study entry	External ocular infection or suspected infection including conjunctivitis and chalazion
No cerebral vascular accident nor myocardial infraction within three months prior to the study entry	Severe intraocular inflammation including uveitis, active rubeosis and endophthalmitis
	History of any other treatment for DME at any time in the past 3 months (such as focal/grid macular photocoagulation, intravitreous or sub-Tenon corticosteroids).
	History of pan-retinal photocoagulation in the past 6 months.
	History of major ocular surgery (including such as vitrectomy, cataract surgery, YAG capsulotomy and any intraocular surgery) within prior 6 months.
	HbA1C ≥10% within two months prior to the study entry
	Uncontrolled blood pressure (defined as systolic ≥160 and/or ≥100mmHg while a patient is a rest)
	History of allergy or hypersensitivity to active drug ranibizumab and any of its excipients, or any study treatment
	Pregnant or breastfeeding

Both eyes of study participants were included only if both of them were eligible for study entry. For safety reasons, both eyes of such participants were not injected with the drug on the same day as part of the initial treatment. In the analysis, both eyes were treated as two independent cases. The study protocols were approved by a certified review board at the Yamaguchi University Hospital (CRB6180002) as well as institutional review boards and ethics committees.

All patients provided written informed consent before their enrolment. In the loading phase, three loading doses of 0.5 mg ranibizumab, an anti-VEGF agent, were administered as part of intravitreal therapy (IVT) (one dose/month, visits 1–3); subsequently, the eyes included in this study were assigned to the responder group or non-responder group depending on the BCVA and/or central foveal retinal thickness (CRT) compared to those before the loading phase (visit 1): responder group, BCVA improvement of ≥5 letters and/or CRT improvement of ≥20%; non-responder group, BCVA improvement of <5 letters and CRT improvement of <20% from visit 1 to visit 4. Non-response in this study was not considered an adverse event.

**Fig 1:**
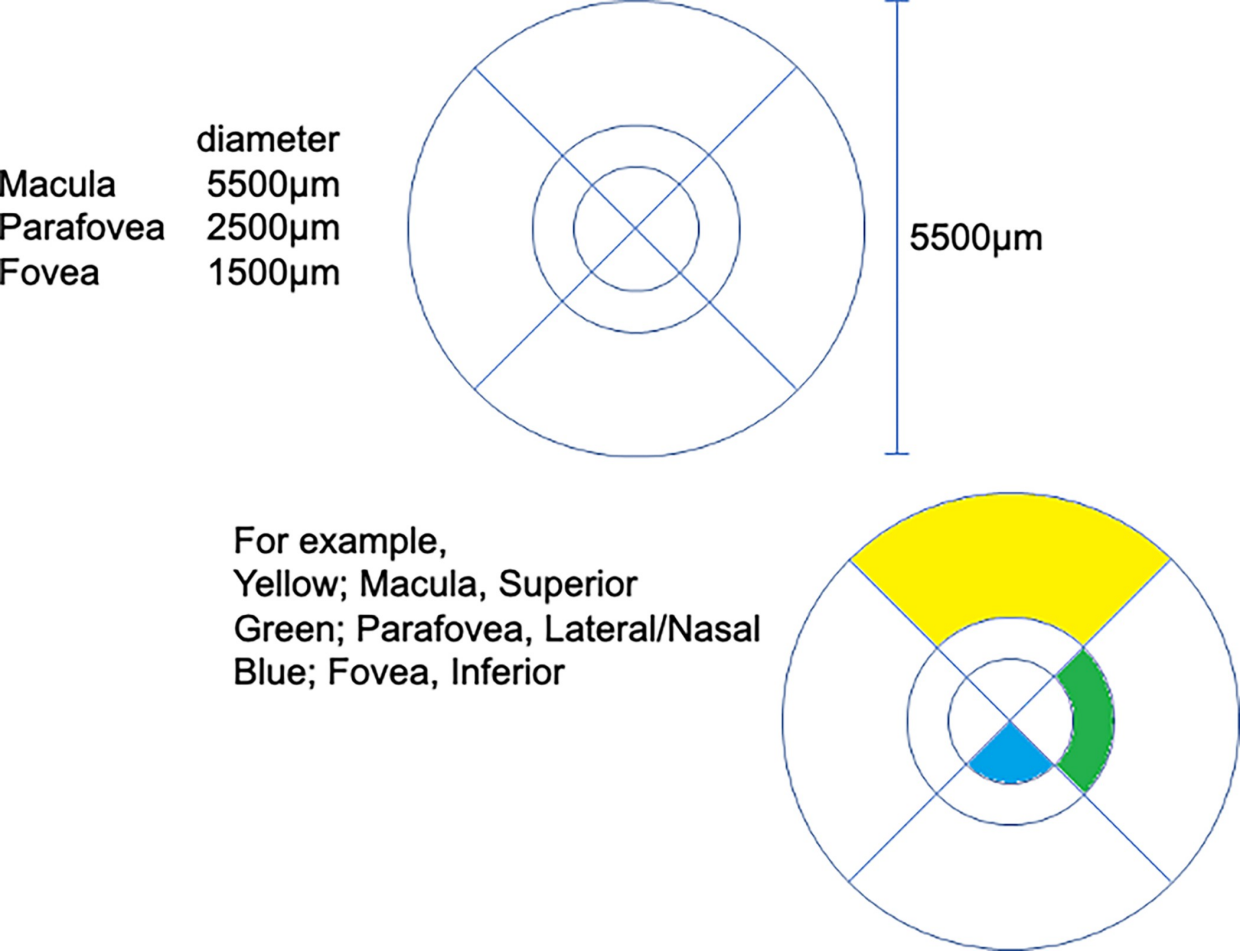
Modified ETDRS grid. (A) Each area of the modified ETDRS grid was showed. (B) As examples, three colors below were displayed. Yellow; Macula, Superior. Green; Parafovea, Lateral/Nasal. Blue; Fovea, Inferior.

We analysed the data from patients who had also undergone IA and/or OCTA in the RELAND study. Age, sex, BCVA, CRT, intraocular pressure (IOP), FA, IA, and OCTA data before ranibizumab IVT and BCVA and CRT data at visit 4 were analysed. The number of MAs in each area of an anatomically modified ETDRS grid (Fig 1) was measured by three retina specialists (MW, RY, and KK).

**Fig 2:**
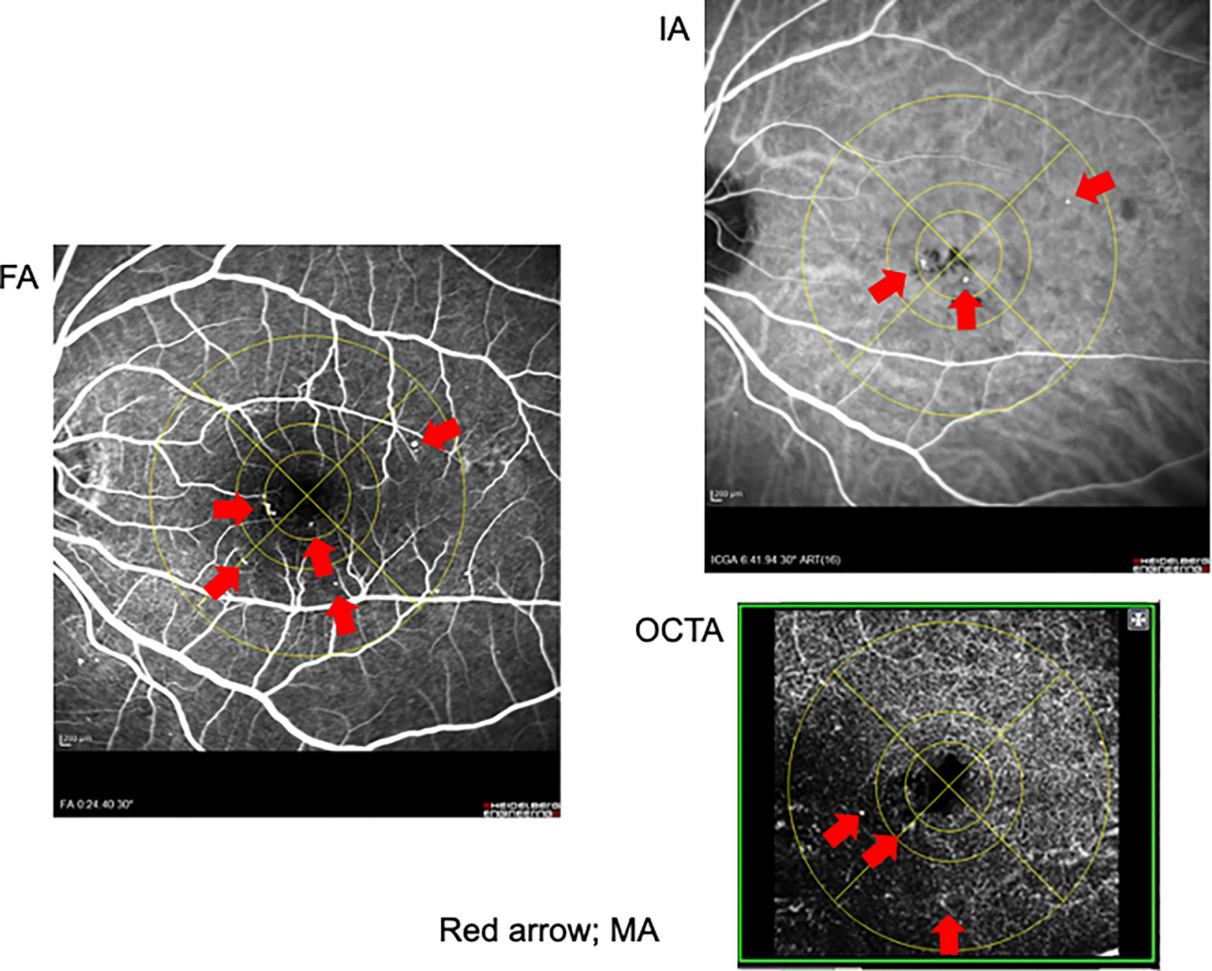
Detected MA of a patient. There are MAs in each the modified ETDRS grid areas. Red arrows indicate MAs.

FA and IA were performed using Heidelberg Retina Angiograph 2 (Heidelberg Engineering). Data of FA in early-phase and data of IA in late-phase were analysed. OCTA was performed using DRI-OCT Triton Plus (Topcon). Scan size was 6.0 mm × 6.0 mm; scan resolution was 320 × 320; repetition was 4 times per line; MAs were analysed in deep capillary plexus layer. To mask the patient’s characteristics, only images of FA, IA, and OCTA were presented to them (Fig 2). On the number of MAs in each area, the inter-rater agreement of the measurers on FA, IA and OCTA were 0.407025, 0.540321, 0.254121, respectively. Regardless of the inter-rater agreement, the average value of the three examiners was used as the MA number.

The primary objective of this study was to determine whether the number of MAs in each macula area of non-responder group is significantly higher than the responder group. The test of the null hypothesis that the number of MAs in both groups is equal was performed by the Welch’s t-test. The test was a two-tailed test with a significance level of 5%. The confidence interval is a two-tailed and the confidence coefficient is 95%. To supplement the primary analysis, the characteristics were also analyzed by the Welch’s t-test and the chi-square test.

## Results

### Characteristics of participants

**Fig 3:**
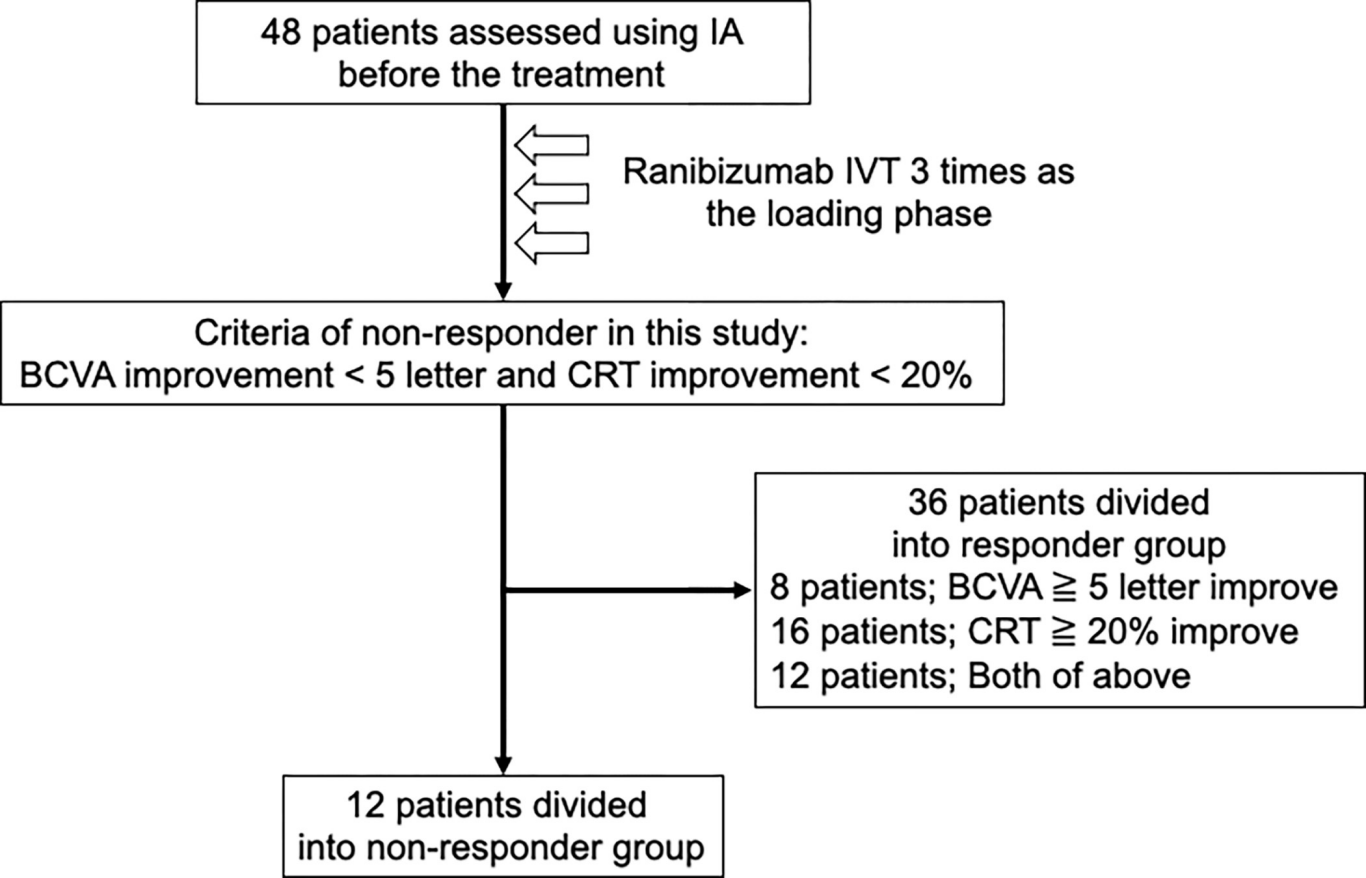
Flow chart. Patients were included in this clinical study, all of whom received ranibizumab IVT.

Forty-eight patients were included in this clinical study, all of whom received ranibizumab IVT; 12 and 36 patients were assigned to the non-responder and responder groups, respectively. Among the 36 patients in the responder group, 8 and 16 patients showed improvements in BCVA (≥5 letters) and CRT (≥20%), respectively, and 12 patients showed improvements in both BCVA and CRT (Fig 3).

The characteristics of the non-responder and responder groups in this study were as follows: age, 69.1 ± 4.3 and 66.8 ± 10.3 years, respectively; male-to-female ratio, 5:7 and 25:11, respectively; BCVA before the loading phase, 69.6 ± 14.3 and 66.7 ± 16.2 letters, respectively; BCVA after the loading phase, 67.3 ± 16.3 and 71.8 ± 14.0 letters, respectively; improvement in BCVA, −2.3 ± 4.4 and 5.1 ± 7.7 letters, respectively; CRT before the loading phase, 373 ± 139 and 389 ± 129, respectively; CRT after the loading phase, 381 ± 140 and 253 ± 111 μm, respectively; improvement in CRT, −4.7 ± 23.3% and 32.9 ± 20.4%, respectively; IOP, 14.7 ± 3.5 and 14.8 ± 3.2 mmHg, respectively (Table 2). There were significant differences in CRT at visit 3 and visit 4 and improvements in BCVA and CRT between the groups. However, there were no significant differences in BCVA and CRT before the loading phase and BCVA at visit 4.

**Table 2:** Characteristics.

	Non-responder	Responder	*p* value
	(N = 12)	(N = 36)	
Age (years; mean ± SD)	69.1 ± 4.3	66.8 ± 10.3	0.485057
Gender (no.; Male: Female)	5: 7	25: 11	0.08519
HbA1c (%)	7.5 ± 1.5	8.2 ± 1.2	0.344098
Pre-treatment BCVA at visit 1	69.6 ± 14.3	66.7 ± 16.2	0.585873
(ETDRS letters; mean ± SD)			
BCVA at visit 2	69.6 ± 14.2	69.2 ± 13.3	0.931332
(ETDRS letters; mean ± SD)			
BCVA at visit 3	69.1 ± 14.5	68.8 ± 13.4	0.956494
(ETDRS letters; mean ± SD)			
BCVA at visit 4	67.3 ± 16.3	71.8 ± 14.0	0.359004
(ETDRS letters; mean ± SD)			
Improvement BCVA	-2.3 ± 4.4	5.1 ± 7.7	0.003007
(ETDRS letters; mean ± SD)			
Pre-treatment CRT at visit1	373 ± 139	389 ± 129	0.725201
(μm; mean ± SD)			
CRT at visit 2 (μm; mean ± SD)	363 ± 145	295 ± 112	0.0968551
CRT at visit 3 (μm; mean ± SD)	387 ± 133	275 ± 129	0.0125176
CRT at visit 4 (μm; mean ± SD)	381 ± 140	253 ± 111	0.0023279
Improvement CRT rate	-4.7 ± 23.3	32.9 ± 20.4	0.00000286569
(%; mean ± SD)			
Pre-treatment IOP	14.7 ± 3.5	14.8 ± 3.2	0.879601
(mmHg; mean ± SD)			

### Relationship between MA and therapeutic response to ranibizumab IVT

There was no significant difference in the number of MAs detected using FA before the loading phase between the non-responder and responder groups (Table 3).

**Table 3:** Detected MA by FA.

		Non-responder	Responder	*p* value
		(N = 12)	(N = 36)	
Macula	Superior	17.0 ± 12.2	14.6 ± 9.0	0.487802
	Inferior	12.4 ± 9.3	9.5 ± 6.6	0.307722
	Lateral	14.5 ± 10.8	10.8 ± 7.5	0.271135
	Nasal	13.2 ± 9.7	9.1 ± 7.2	0.183823
Parafovea	Superior	2.5 ± 2.3	1.7 ± 1.8	0.240609
	Inferior	1.7 ± 1.4	1.4 ± 1.7	0.479289
	Lateral	1.4 ± 1.5	2.3 ± 3.0	0.215847
	Nasal	1.6 ± 1.2	1.6 ± 1.8	0.942946
Fovea	Superior	1.5 ± 1.5	0.8 ± 1.0	0.160108
	Inferior	1.0 ± 1.0	0.9 ± 1.7	0.755671
	Lateral	1.3 ± 1.3	0.7 ± 0.7	0.142024
	Nasal	1.5 ± 2.2	0.5 ± 0.9	0.191712

Data are means ± SD, *p* value were calculated using Welch’s t-test.

The number of MAs detected using IA was similar between the groups (Table 4).

**Table 4:** Detected MA by IA.

		Non-responder	Responder	*p* value
		(N = 12)	(N = 36)	
Macula	Superior	12.3 ± 10.2	7.8 ± 7.0	0.158274
	Inferior	8.1 ± 7.1	6.4 ± 6.3	0.427039
	Lateral	9.0 ± 7.0	6.3 ± 5.8	0.223681
	Nasal	12.5 ± 10.6	5.7 ± 5.7	0.0477773
Parafovea	Superior	2.1 ± 2.3	1.6 ± 2.0	0.464580
	Inferior	0.9 ± 0.9	1.5 ± 2.1	0.236424
	Lateral	1.0 ±1.1	1.4 ± 1.8	0.451470
	Nasal	1.1 ± 1.1	1.2 ± 1.6	0.844735
Fovea	Superior	1.0 ± 0.7	0.6 ± 0.8	0.0919663
	Inferior	0.5 ± 0.8	0.7 ± 1.6	0.549018
	Lateral	0.7 ± 1.0	0.3 ± 0.7	0.175130
	Nasal	1.4 ± 2.1	0.4 ± 0.7	0.0391342

Data are means ± SD, *p* value were calculated using Welch’s t-test.

Surprisingly, the non-responder group showed a higher number of MAs, mainly in the nasal area, than did the responder group. In particular, there were significant differences in the nasal macula (non-responder group, 12.5 ± 10.6; responder group, 5.7 ± 5.7, *P* < 0.05) and nasal fovea (non-responder group, 1.4 ± 2.1; responder group, 0.4 ± 0.7, *P* < 0.05). There was no significant difference between the groups regarding the number of MAs detected by OCTA (Table 5).

**Table 5:** Detected MA by OCTA.

		Non-responder	Responder	*p* value
		(N = 12)	(N = 29)	
Macula	Superior	6.3 ± 3.8	5.3 ± 4.4	0.424897
	Inferior	5.3 ± 3.9	6.2 ± 3.8	0.540926
	Lateral	4.5 ± 3.3	5.5 ± 2.9	0.41803
	Nasal	4.3 ± 3.4	4.2 ± 3.0	0.988004
Parafovea	Superior	1.5 ± 1.7	1.4 ± 1.2	0.655697
	Inferior	0.9 ± 1.0	1.5 ± 1.0	0.141042
	Lateral	0.9 ± 0.9	1.2 ± 1.2	0.430182
	Nasal	0.9 ± 0.7	1.1 ± 0.9	0.546775
Fovea	Superior	0.4 ± 0.7	0.6 ± 0.6	0.617404
	Inferior	0.5 ± 0.5	0.5 ± 0.9	0.970334
	Lateral	0.4 ± 0.5	0.6 ± 0.7	0.424587
	Nasal	0.5 ± 1.1	0.4 ± 0.6	0.997254

Data are means ± SD, *p* value were calculated using Welch’s t-test.

## Discussion

**Fig 4:**
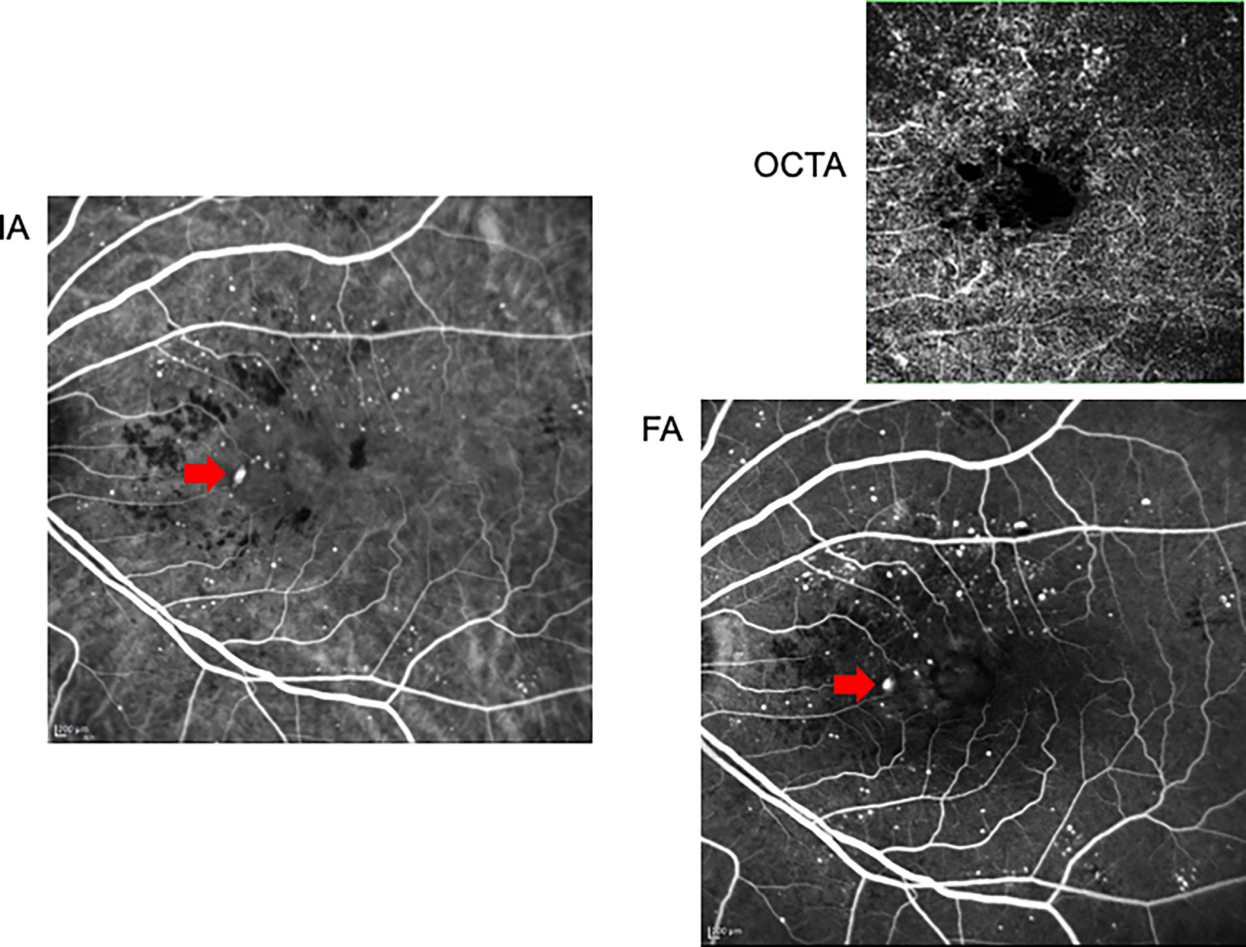
Detected MA of a non-responder case. 68-years-old male. There was a large MA (red allow) surrounded blockings by hard exudates in nasal macula area. IA and FA could detect the MA; OCTA could not detect.

**Fig 5:**
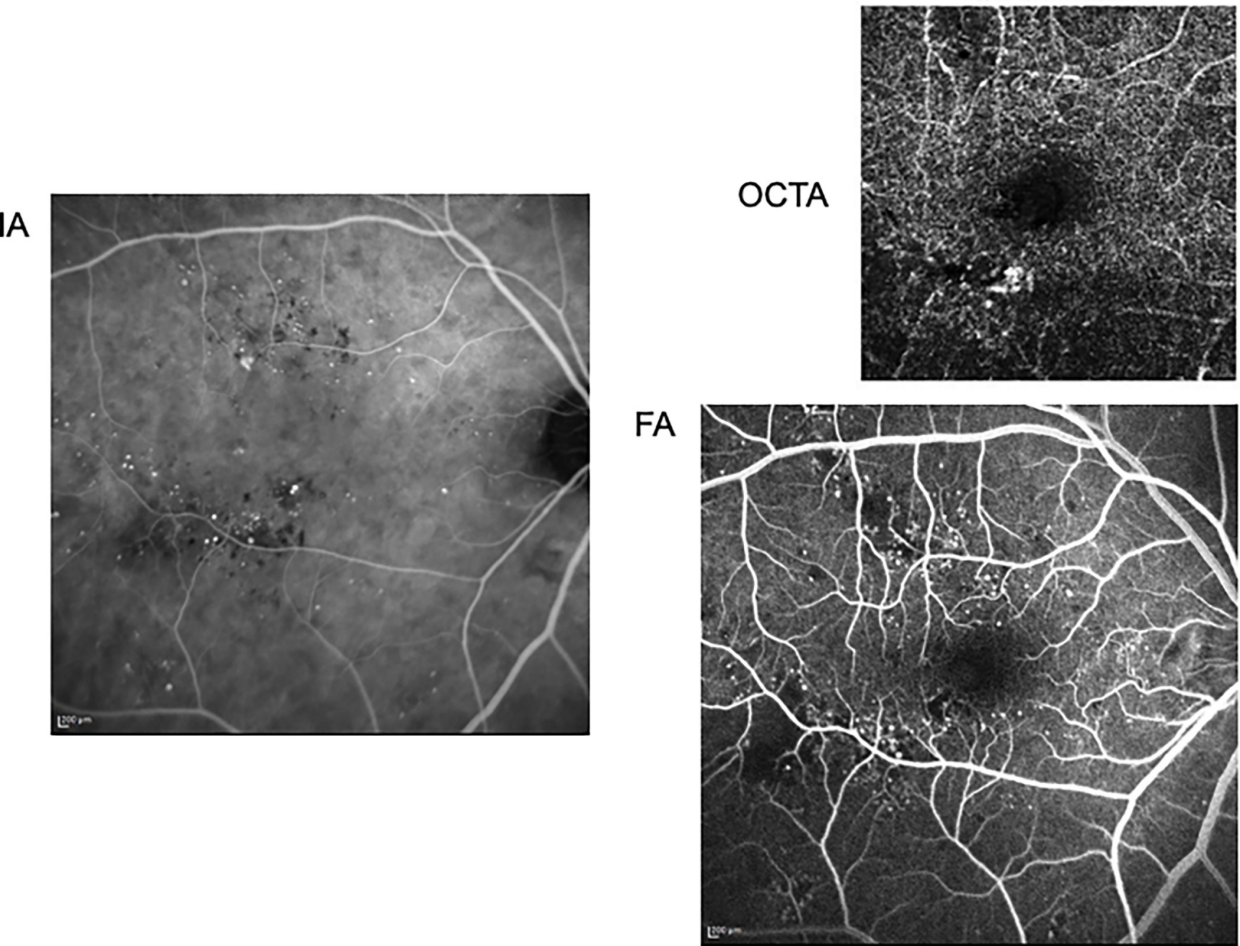
Detected MA of a responder case. 67-years-old female. There was less MAs in nasal area in spite of many MAs in superior and inferior area. Not only FA and OCTA but also IA could not detect noticeable MAs in nasal area.

Anti-VEGF therapy for DME improves visual acuity and anatomical macular changes [25–29]. However, all patients with DME do not always respond to anti-VEGF therapy. Therefore, it is necessary to clarify the factors influencing the resistance to anti-VEGF therapy in patients with DME. MA involves the out-punching of retinal capillaries that are weakened by the loss of pericytes, which results in retinal focal haemorrhage [6, 7]. The presence of MAs is a predictor of DME development and progression [8, 10]. MAs detected by angiographies in patients with DME also contribute to the clinical response to anti-VEGF therapy [30, 31]. Indeed, the use of laser photocoagulation for leaking MAs was found to be effective for the treatment DME [1]. In this study, we examined the distribution of MAs by using multiple angiography techniques (FA, IA, and OCTA) and then investigated the relationship between the presence or distribution of MAs and the response to anti-VEGF therapy. Notably, MAs detected by IA in the nasal area contributed to resistance of the patients to three anti-VEGF loading doses. IA appears to accumulate at the permeable MA that is responsible for macular oedema [32]. As a supplement, there was no significant difference of MAs in nasal parafovea area between the two groups. The nasal parafoveal area might be too narrow to allow statistical significance. Whereas, MAs detected by IA especially contribute to macular oedema responsible for DME because IA-guided navigated focal laser photocoagulation is effective for the treatment of DME [32]. These results suggest that the presence of MAs detected by IA in the nasal area of the macula was associated with resistance to three anti-VEGF loading doses. We presented the multimodal images of representative cases in both group (Figs 4 and 5).

MAs are generally the earliest signs of clinically visible retinal damage and are a hallmark of DME. Accurate identification is important because their distribution and number provide prognostic information about the severity of DM and are also clinically relevant as a guide for the treatment of DME [18]. Indeed, the reduction of macular oedema is associated with the non-perfusion area, neovascularization, and number of MAs [33]. Macular capillary blood flow in patients with DME was found to be significantly lower than that in those with non-diabetes [34]. The nasal-temporal asymmetry of blood flow in the macular capillaries was significantly high in patients with DME. Furthermore, oxygen saturation is low on the nasal side of the macula [25]. The blood flow on the nasal side of the macula may be important for maintaining macular function and is highly sensitive to small changes in blood flow or oxygen saturation. MAs also localise to the nasal side of the macula and may interfere with normal nerve signalling in the papillary macular nerve fibre bundle, which results in indirect visual loss [35]. After the administration of three loading doses of the anti-VEGF agent, MAs detected on the nasal side of the macula using IA were not responsive to anti-VEGF therapy. The presence of large MA detected by IA in the nasal macula indicates that the DME condition is physiologically and anatomically severe, and such cases are likely to resist anti-VEGF therapy.

Accurate delineation of MA distribution, that is, location and number, is important for obtaining prognostic information about the severity of DME and for planning the treatment of DME [12]. We investigated whether there were differences in the distribution of MAs between the responder and non-responder groups, using FA, IA, and OCTA. Interestingly, the MAs detected using IA, especially those on the nasal side of the macula, were predictive of resistance to three anti-VEGF loading doses, while those detected using FA and OCTA do not have predictive value in this regard. Theoretically, considering the molecular size, the sensitivity to detect MA is highest in the order of FA, IA, and OCTA. Because the molecular weight unit is 332, 775 and 64500, respectively. In this study, the total number of MAs in Tables 3–5 was consistent with the order. Also, we selectively analysed MAs in deep capillary plexus layer. Moreover, there were not monitoring centre to check the quality of the images. The noise in OCTA might have masked MA detection. Therefore, the detection sensitivities of OCTA in this study tended lower than other reports. Whereas, IA is useful for identifying leaking spots or larger capillaries in patients with DME [32, 36]. These previous data indicate that IA could characteristically help visualise MAs because of its low detection sensitivity. There is a report that the improvement of DME by anti-VEGF therapy correlated with the size of MAs [37]. Therefore, small MA may respond well to anti-VEGF therapy. In this study, MAs detected on the nasal macula using IA was less than FA and OCTA in the responder group. This was because the responder group had many small MAs of detectable size for FA but not for IA. Moreover, MAs in the nasal macula is unlikely to develop for the reasons of the blood flow and oxygen saturation. However, when MAs develop large enough to be detected by IA, they may affect the effectiveness of treatment. Therefore, we assumed that IA, but not FA and OCT, is useful in predicting the response to anti-VEGF therapy. The number of cases might be too small to be significantly different in FA and OCTA. Whereas, MAs detected using IA are also detected at the same site in FA and OCTA. Also, Therefore, more studies with a larger number of cases are needed to describe whether such Mas can be detected in FA and OCTA.

We have shown that the presence of MA on the nasal side of the macula detected using IA may be a biomarker of resistance to anti-VEGF therapy among patients with DME. The disruption of the blood-retinal barrier by VEGF contributes to the pathogenesis of DME [38]. Indeed, anti-VEGF agents are used as the primary treatment for DME, and these agents effectively improve macular oedema and vision in most patients with DME [25–29]. It is important to identify factors that predict resistance to anti-VEGF agents because the cost of anti-VEGF drugs is a major burden for patients with DME. There are many predictors of response to anti-VEGF therapy for DME, including pre-treatment CRT, HbA1c level [27], and presence of subretinal fluid, intraretinal cysts, and renal disease [28]. We focused on the relationship between the distribution of MAs and the reactivity of the treatment. Local retinal circulation is thought to have a great influence on the formation and distribution of MAs. Further investigation is needed to correlate the distribution of MAs between these predictors.

Among participant characteristics, there were no significant differences in BCVA from visit 1 to visit 4 (Table 2). There are studies in which some cases of DME did not show improvement despite the administration of intensive anti-VEGF therapy [4, 5]. Although criteria for therapeutic response to DME varied, these studies reported resistance to anti-VEGF therapy in 18–30% of DME eyes. Consistent with the data of previous studies, 25% (12/48) of DME eyes showed resistance in this study. Our results can also be explained by the fact that the pre-treatment BCVA was relatively better that that in other clinical studies [28, 39] and that the responder group showed lesser BCVA improvement than CRT improvement (Fig 3). The limitations of the study were the exploratory decision of the reactivity criteria and the unevenness of improvement in BCVA and CRT in the responder group.

In conclusion, detection on MAs in the nasal macular area using IA before anti-VEGF therapy of treatment naïve DME might indicate treatment resistance. We recommend the confirmation of the presence of MAs in the nasal macular area using IA to predict the therapeutic response to anti-VEGF therapy in treatment naïve DME eyes.

## Supporting information

S1 Table Minimal data set of characteristics. Sex, age, HbA1c, CRT, BCVA of each group are showed.
(XLSX)

S2 Table Minimal data set of the number of detected MAs by FA. The number of MAs counted by three measurers in each area on FA.
(XLSX)

S3 Table Minimal data set of the number of detected MAs by IA. The number of MAs counted by three measurers in each area on IA.
(XLSX)

S4 Table Minimal data set of the number of detected MAs by OCTA. The number of MAs counted by three measurers in each area on OCTA.
(XLSX)

## References

[1] Photocoagulation for Diabetic Macular Edema: Early Treatment Diabetic Retinopathy Study Report Number 1 Early Treatment Diabetic Retinopathy Study Research Group. Archives of Ophthalmology. 1985;103(12):1796–806. doi: 10.1001/archopht.1985.01050120030015 2866759

[2] Yoshida S, Murakami T, Nozaki M, Suzuma K, Baba T, Hirano T, et al. Review of clinical studies and recommendation for a therapeutic flow chart for diabetic macular edema. Graefes Arch Clin Exp Ophthalmol. 2020. Epub 2020/10/01. doi: 10.1007/s00417-020-04936-w . 32997288

[3] Wells JA, Glassman AR, Ayala AR, Jampol LM, Bressler NM, Bressler SB, et al. Aflibercept, Bevacizumab, or Ranibizumab for Diabetic Macular Edema: Two-Year Results from a Comparative Effectiveness Randomized Clinical Trial. Ophthalmology. 2016;123(6):1351–9. Epub 2016/03/05. doi: 10.1016/j.ophtha.2016.02.022 ; PubMed Central PMCID: PMC4877252. 26935357 PMC4877252

[4] Shimura M, Yasuda K, Motohashi R, Kotake O, Noma H. Aqueous cytokine and growth factor levels indicate response to ranibizumab for diabetic macular oedema. Br J Ophthalmol. 2017;101(11):1518–23. Epub 2017/03/09. doi: 10.1136/bjophthalmol-2016-309953 . 28270488

[5] Wells JA, Glassman AR, Ayala AR, Jampol LM, Aiello LP, Antoszyk AN, et al. Aflibercept, bevacizumab, or ranibizumab for diabetic macular edema. N Engl J Med. 2015;372(13):1193–203. Epub 2015/02/19. doi: 10.1056/NEJMoa1414264 ; PubMed Central PMCID: PMC4422053. 25692915 PMC4422053

[6] Garner A. Histopathology of diabetic retinopathy in man. Eye. 1993;7(2):250–3. doi: 10.1038/eye.1993.58 7607344

[7] Stitt AW, Gardiner TA, Archer DB. Histological and ultrastructural investigation of retinal microaneurysm development in diabetic patients. Br J Ophthalmol. 1995;79(4):362–7. Epub 1995/04/01. doi: 10.1136/bjo.79.4.362 ; PubMed Central PMCID: PMC505103. 7742285 PMC505103

[8] Pappuru RKR, Ribeiro L, Lobo C, Alves D, Cunha-Vaz J. Microaneurysm turnover is a predictor of diabetic retinopathy progression. Br J Ophthalmol. 2019;103(2):222–6. Epub 2018/04/28. doi: 10.1136/bjophthalmol-2018-311887 ; PubMed Central PMCID: PMC6362804. 29699981 PMC6362804

[9] Wharton H, Dodson PM, Gibson J. The significance of microaneurysms and haemorrhages within one optic disc diameter of the fovea. European Journal of Ophthalmology. 2010;20(3).

[10] Haritoglou C, Kernt M, Neubauer A, Gerss J, Oliveira CM, Kampik A, et al. MICROANEURYSM FORMATION RATE AS A PREDICTIVE MARKER FOR PROGRESSION TO CLINICALLY SIGNIFICANT MACULAR EDEMA IN NONPROLIFERATIVE DIABETIC RETINOPATHY. RETINA. 2014;34(1):157–64. doi: 10.1097/IAE.0b013e318295f6de -201401000-00022. 23792485

[11] Reznicek L, Kernt M, Haritoglou C, Ulbig M, Kampik A, Neubauer AS. Correlation of leaking microaneurysms with retinal thickening in diabetic retinopathy. Int J Ophthalmol. 2011;4(3):269–71. Epub 2011/01/01. doi: 10.3980/j.issn.2222-3959.2011.03.11 ; PubMed Central PMCID: PMC3340824. 22553659 PMC3340824

[12] Treatment techniques and clinical guidelines for photocoagulation of diabetic macular edema. Early Treatment Diabetic Retinopathy Study Report Number 2. Early Treatment Diabetic Retinopathy Study Research Group. Ophthalmology. 1987;94(7):761–74. Epub 1987/07/01. doi: 10.1016/s0161-6420(87)33527-4 . 3658348

[13] Rand LI, Prud’homme GJ, Ederer F, Canner PL. Factors influencing the development of visual loss in advanced diabetic retinopathy. Diabetic Retinopathy Study (DRS) Report No. 10. Invest Ophthalmol Vis Sci. 1985;26(7):983–91. Epub 1985/07/01. . 2409053

[14] Novotny HR, Alvis DL. A method of photographing fluorescence in circulating blood in the human retina. Circulation. 1961;24:82–6. Epub 1961/07/01. doi: 10.1161/01.cir.24.1.82 . 13729802

[15] Kogure K, David NJ, Yamanouchi U, Choromokos E. Infrared absorption angiography of the fundus circulation. Arch Ophthalmol. 1970;83(2):209–14. Epub 1970/02/01. doi: 10.1001/archopht.1970.00990030211015 . 4983539

[16] Saito T, Komatsu Y, Mori S, Deguchi T, Koyama I, Yoneya S. [A study of serum protein fraction binding to indocyanine green (ICG) by combined method of immunoelectrophoresis and ICG fundus videosystem]. Nippon Ganka Gakkai Zasshi. 1996;100(8):617–23. Epub 1996/08/01. . 8810238

[17] Gao SS, Jia Y, Zhang M, Su JP, Liu G, Hwang TS, et al. Optical Coherence Tomography Angiography. Invest Ophthalmol Vis Sci. 2016;57(9):Oct27–36. Epub 2016/07/15. doi: 10.1167/iovs.15-19043 ; PubMed Central PMCID: PMC4968919. 27409483 PMC4968919

[18] Stattin M, Haas A-M, Ahmed D, Stolba U, Graf A, Krepler K, et al. Detection rate of diabetic macular microaneurysms comparing dye-based angiography and optical coherence tomography angiography. Scientific Reports. 2020;10(1):16274. doi: 10.1038/s41598-020-73516-z 33005009 PMC7530679

[19] Castro Farías D, Matsui Serrano R, Bianchi Gancharov J, de Dios Cuadras U, Sahel J, Graue Wiechers F, et al. Indocyanine green angiography for identifying telangiectatic capillaries in diabetic macular oedema. British Journal of Ophthalmology. 2020;104(4):509–13. doi: 10.1136/bjophthalmol-2019-314355 31358497

[20] Parrulli S, Corvi F, Cozzi M, Monteduro D, Zicarelli F, Staurenghi G. Microaneurysms visualisation using five different optical coherence tomography angiography devices compared to fluorescein angiography. British Journal of Ophthalmology. 2021;105(4):526–30. doi: 10.1136/bjophthalmol-2020-316817 32527718 PMC8005788

[21] Hasegawa N, Nozaki M, Takase N, Yoshida M, Ogura Y. New Insights Into Microaneurysms in the Deep Capillary Plexus Detected by Optical Coherence Tomography Angiography in Diabetic Macular Edema. Invest Ophthalmol Vis Sci. 2016;57(9):Oct348-55. Epub 2016/07/15. doi: 10.1167/iovs.15-18782 . 27409492

[22] Cui Y, Zhu Y, Wang JC, Lu Y, Zeng R, Katz R, et al. Imaging Artifacts and Segmentation Errors With Wide-Field Swept-Source Optical Coherence Tomography Angiography in Diabetic Retinopathy. Translational Vision Science & Technology. 2019;8(6):18–. doi: 10.1167/tvst.8.6.18 31772829 PMC6859832

[23] Lee J, Moon BG, Cho AR, Yoon YH. Optical Coherence Tomography Angiography of DME and Its Association with Anti-VEGF Treatment Response. Ophthalmology. 2016;123(11):2368–75. Epub 2016/10/25. doi: 10.1016/j.ophtha.2016.07.010 . 27613201

[24] Mori K, Yoshida S, Kobayashi Y, Ishikawa K, Nakao S, Hisatomi T, et al. Decrease in the number of microaneurysms in diabetic macular edema after anti-vascular endothelial growth factor therapy: implications for indocyanine green angiography-guided detection of refractory microaneurysms. Graefes Arch Clin Exp Ophthalmol. 2020;258(4):735–41. Epub 2020/01/22. doi: 10.1007/s00417-020-04608-9 . 31960130

[25] Do DV, Schmidt-Erfurth U, Gonzalez VH, Gordon CM, Tolentino M, Berliner AJ, et al. The DA VINCI Study: phase 2 primary results of VEGF Trap-Eye in patients with diabetic macular edema. Ophthalmology. 2011;118(9):1819–26. Epub 2011/05/07. doi: 10.1016/j.ophtha.2011.02.018 . 21546089

[26] Heier JS, Korobelnik JF, Brown DM, Schmidt-Erfurth U, Do DV, Midena E, et al. Intravitreal Aflibercept for Diabetic Macular Edema: 148-Week Results from the VISTA and VIVID Studies. Ophthalmology. 2016;123(11):2376–85. Epub 2016/10/25. doi: 10.1016/j.ophtha.2016.07.032 . 27651226

[27] Massin P, Bandello F, Garweg JG, Hansen LL, Harding SP, Larsen M, et al. Safety and efficacy of ranibizumab in diabetic macular edema (RESOLVE Study): a 12-month, randomized, controlled, double-masked, multicenter phase II study. Diabetes Care. 2010;33(11):2399–405. Epub 2010/10/29. doi: 10.2337/dc10-0493 ; PubMed Central PMCID: PMC2963502. 20980427 PMC2963502

[28] Mitchell P, Bandello F, Schmidt-Erfurth U, Lang GE, Massin P, Schlingemann RO, et al. The RESTORE study: ranibizumab monotherapy or combined with laser versus laser monotherapy for diabetic macular edema. Ophthalmology. 2011;118(4):615–25. Epub 2011/04/05. doi: 10.1016/j.ophtha.2011.01.031 . 21459215

[29] Nguyen QD, Brown DM, Marcus DM, Boyer DS, Patel S, Feiner L, et al. Ranibizumab for diabetic macular edema: results from 2 phase III randomized trials: RISE and RIDE. Ophthalmology. 2012;119(4):789–801. Epub 2012/02/15. doi: 10.1016/j.ophtha.2011.12.039 . 22330964

[30] Kaizu Y, Nakao S, Wada I, Arima M, Yamaguchi M, Ishikawa K, et al. Microaneurysm Imaging Using Multiple En Face OCT Angiography Image Averaging: Morphology and Visualization. Ophthalmology Retina. 2020;4(2):175–86. doi: 10.1016/j.oret.2019.09.010 31753811

[31] Pongsachareonnont P, Charoenphol P, Hurst C, Somkijrungroj T. The Effect of Anti-Vascular Endothelial Growth Factor on Retinal Microvascular Changes in Diabetic Macular Edema Using Swept-Source Optical Coherence Tomography Angiography. Clin Ophthalmol. 2020;14:3871–80. Epub 2020/11/26. doi: 10.2147/OPTH.S270410 ; PubMed Central PMCID: PMC7678686. 33235428 PMC7678686

[32] Nozaki M, Kato A, Yasukawa T, Suzuki K, Yoshida M, Ogura Y. Indocyanine green angiography-guided focal navigated laser photocoagulation for diabetic macular edema. Jpn J Ophthalmol. 2019;63(3):243–54. Epub 2019/02/27. doi: 10.1007/s10384-019-00662-x . 30806869

[33] Xue K, Yang E, Chong NV. Classification of diabetic macular oedema using ultra-widefield angiography and implications for response to anti-VEGF therapy. British Journal of Ophthalmology. 2017;101(5):559–63. doi: 10.1136/bjophthalmol-2016-308704 27531355

[34] Hudson C, Flanagan J, Turner G, Chen H, Rawji M, McLeod D. Exaggerated relative nasal-temporal asymmetry of macular capillary blood flow in patients with clinically significant diabetic macular oedema. The British journal of ophthalmology. 2005;89:142–6. doi: 10.1136/bjo.2003.037317 15665341 PMC1772510

[35] Konieczka K, Bojinova RI, Valmaggia C, Schorderet DF, Todorova MG. Preserved functional and structural integrity of the papillomacular area correlates with better visual acuity in retinitis pigmentosa. Eye (Lond). 2016;30(10):1310–23. Epub 2016/08/06. doi: 10.1038/eye.2016.136 ; PubMed Central PMCID: PMC5129858. 27494084 PMC5129858

[36] Ogura S, Yasukawa T, Kato A, Kuwayama S, Hamada S, Hirano Y, et al. Indocyanine Green Angiography-Guided Focal Laser Photocoagulation for Diabetic Macular Edema. Ophthalmologica. 2015;234(3):139–50. Epub 2015/09/24. doi: 10.1159/000437360 . 26393771

[37] Sugimoto M, Ichio A, Mochida D, Tenma Y, Miyata R, Matsubara H, et al. Multiple Effects of Intravitreal Aflibercept on Microvascular Regression in Eyes with Diabetic Macular Edema. Ophthalmology Retina. 2019;3(12):1067–75. doi: 10.1016/j.oret.2019.06.005 31446029

[38] Zhang X, Zeng H, Bao S, Wang N, Gillies MC. Diabetic macular edema: new concepts in patho-physiology and treatment. Cell Biosci. 2014;4:27. Epub 2014/06/24. doi: 10.1186/2045-3701-4-27 ; PubMed Central PMCID: PMC4046142. 24955234 PMC4046142

[39] Brown DM, Nguyen QD, Marcus DM, Boyer DS, Patel S, Feiner L, et al. Long-term Outcomes of Ranibizumab Therapy for Diabetic Macular Edema: The 36-Month Results from Two Phase III Trials: RISE and RIDE. Ophthalmology. 2013;120(10):2013–22. doi: 10.1016/j.ophtha.2013.02.034 23706949

